# HPOAnnotator: improving large-scale prediction of HPO annotations by low-rank approximation with HPO semantic similarities and multiple PPI networks

**DOI:** 10.1186/s12920-019-0625-1

**Published:** 2019-12-23

**Authors:** Junning Gao, Lizhi Liu, Shuwei Yao, Xiaodi Huang, Hiroshi Mamitsuka, Shanfeng Zhu

**Affiliations:** 1 School of Computer Science and Shanghai Key Laboratory of Intelligent Information Processing, Fudan University, 220 Handan Road, Shanghai, 200433 China; 2 School of Computing and Mathematics, Charles Sturt University, Elizabeth Mitchell Dr, Albury, NSW 2640 Australia; 3 Bioinformatics Center, Institute for Chemical Research, Kyoto University, Kashiwada Gokasho, Uji, Kyoto, 611-0011 Japan; 4 Department of Computer Science, Aalto University, Konemiehentie 2, Espoo, 02150 Finland; 5 Shanghai Institute of Artificial Intelligence Algorithms and ISTBI, Fudan University, Shanghai, 200433 China; 6 Key Laboratory of Computational Neuroscience and Brain-Inspired Intelligence (Fudan University), Ministry of Education, Shanghai, China

**Keywords:** Low-rank approximation, Human phenotype ontology, Protein-protein interaction networks, Hierarchical structure

## Abstract

**Background:**

As a standardized vocabulary of phenotypic abnormalities associated with human diseases, the Human Phenotype Ontology (HPO) has been widely used by researchers to annotate phenotypes of genes/proteins. For saving the cost and time spent on experiments, many computational approaches have been proposed. They are able to alleviate the problem to some extent, but their performances are still far from satisfactory.

**Method:**

For inferring large-scale protein-phenotype associations, we propose HPOAnnotator that incorporates multiple Protein-Protein Interaction (PPI) information and the hierarchical structure of HPO. Specifically, we use a dual graph to regularize Non-negative Matrix Factorization (NMF) in a way that the information from different sources can be seamlessly integrated. In essence, HPOAnnotator solves the sparsity problem of a protein-phenotype association matrix by using a low-rank approximation.

**Results:**

By combining the hierarchical structure of HPO and co-annotations of proteins, our model can well capture the HPO semantic similarities. Moreover, graph Laplacian regularizations are imposed in the latent space so as to utilize multiple PPI networks. The performance of HPOAnnotator has been validated under cross-validation and independent test. Experimental results have shown that HPOAnnotator outperforms the competing methods significantly.

**Conclusions:**

Through extensive comparisons with the state-of-the-art methods, we conclude that the proposed HPOAnnotator is able to achieve the superior performance as a result of using a low-rank approximation with a graph regularization. It is promising in that our approach can be considered as a starting point to study more efficient matrix factorization-based algorithms.

## Background

Phenotypes refer to observable physical or biological traits of an organism. Revealing the relationships between genes/proteins and their related phenotypes is one of the main objectives of genetics in the post-genome era [1–3]. The Human Phenotype Ontology (HPO) [4] is a standardized vocabulary for describing the phenotypic abnormalities associated with human diseases [5]. Being initially populated by using databases of human genes and genetic disorders such as OMIM [6], Orphanet [7] and DECIPHER [8], HPO was later expanded by using literature curation [9]. At present, only small quantities of human protein-coding genes (∼3500) have HPO annotations. It is, however, believed that a large number of currently unannotated genes/proteins are related to disease phenotypes. Therefore, it is critical to predict genes/protein-HPO associations by using accurate computational methods.

Currently, HPO contains four sub-ontologies: Organ abnormality, Mode of inheritance, Clinical modifier, and Mortality/Aging. As the main sub-ontology, Organ abnormality describes clinical abnormalities whose first- level children are formed by terms like abnormality of a skeletal system. The Mode of inheritance describes inheritance patterns of phenotypes and contains terms such as Autosomal dominant. The Clinical modifier contains classes that describe typical modifiers of clinical symptoms such as those triggered by carbohydrate ingestion. For Mortality/Aging, it describes the age of death by terms like Death in childhood and Sudden death. The Organ abnormality, Mode of inheritance, Clinical modifier, and Mortality/Aging have ∼12000, 28, 100, and 8 terms, respectively.

**Fig. 1:**
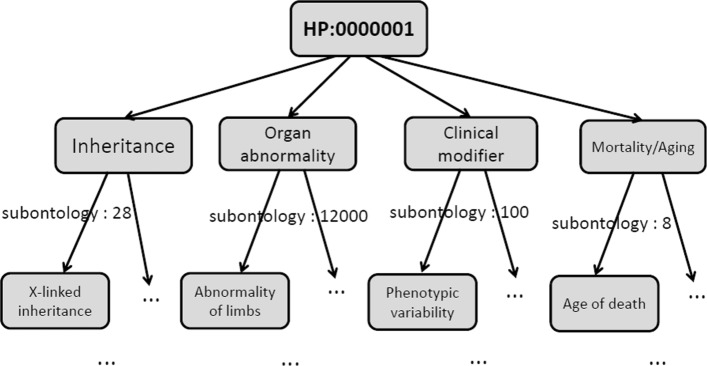
An example of the HPO hierarchical tree. All parent-child relationships in HPO represent “is-a” relationships. X-linked inheritance, Abnormality of limbs, Phenotypic variability, and Age of death are examples for sub-ontologies Mode of inheritance, Organ abnormality, Clinical modifier, and Mortality/Aging, respectively

The annotations between genes/proteins and HPO terms are very sparse. Specifically, 284621 annotations are for 3459 proteins and 6407 HPO terms with the sparsity of 1.2%. Meanwhile, the annotation growth by time, for example, is about 5%, with adding only 14820 annotations as new ones between June 2017 to December 2017. Since genes/proteins are annotated with multiple HPO terms, the prediction can be regarded as a problem of multi-label predictions. Differing from this, HPO terms, however, form a hierarchical structure. This implies that once a gene/protein is labeled with one HPO term, it should also be labeled with all of its ancestors of this particular HPO term. In other words, when a gene/protein is not labeled with an HPO term, it should not be labeled with all of its descendants, either. That is, general terms are located at the top of the HPO structure, with the term specificity increasing from the root to the leaves. Figure 1 shows a real example of an HPO hierarchical structure (i.e., Directed Acyclic Graph, DAG) and the scale of sub-ontologies.

The existing computational approaches for HPO annotation prediction can be divided into two categories, namely feature-based and network-based methods. The feature-based approaches use gene/protein information as the features to predict its annotations for a query gene/protein. For sparse and noisy data, the incorporation of auxiliary information into original input data generally helps to improve predictive performance. One of these methods, learning to rank, has been demonstrated the superior performance in GO annotation prediction [10], for example. Compared with GO annotations, HPO annotations are, however, more reliable and stable. In addition, the sparseness of HPO annotations is much less than that of GO annotations, with focusing on human proteins and terms under Organ abnormality only. Nevertheless, few existing feature-based models take into consideration HPO information, e.g., the hierarchical structure and co-occurrence of HPO terms. The network-based approaches are more prevalent at present. Usually, multiple networks are integrated into a new large-scale network in order to improve the prediction in these approaches such as random-walk [11] and weighted score computation [12]. However, network-based approaches cannot perform well for sparse data. This is because of disconnected nodes that are commonly encountered in real-world graphs, particulary for sparse data, even though they can be related to each other.

Prediction of the annotations between genes/proteins and HPO terms can be grouped into two categorises: 1) pair prediction, which predicts the missing HPO annotations of existing proteins, and 2) prediction of new proteins, which annotates HPO terms to the totally unannotated proteins. Most existing work belong to the latter category, but few are for the former. To narrow this gap, we focus on the first category in this paper, which is also a famous task in the CAFA challenge. Existing methods for the first category have four major limitations. First, the hierarchy of HPO is completely ignored. The hierarchical structure poses a formidable challenge to a prediction: a model needs to evaluate the associations between a protein and all of its related phenotypes from the deeper levels to the root in the hierarchy. Second, the existing methods do not make full use of the potentials of Protein-Protein Interaction (PPI) networks. For example, a PPI network is modeled in the original annotation space in their models, which may not extract the information effectively. Moreover, multiple PPI networks may be derived from different sources, resulting in the data fusion. Third, only a few known associations are available for training. So they are extremely unbalanced. Specifically, more than half of the terms in HPO are used to annotate zero or only one protein. As a result, such a drastic sparsity makes prediction more challenging. Finally, existing methods usually study the sub-ontologies independently without considering the co-annotations of HPO terms. However, co-annotations are quite common in annotations. It is likely that they help improve prediction results.

To address the above four problems, we apply matrix factorization to approximate a protein-HPO annotation matrix by two factorized low-rank matrices. As such, the latent factors that underlie the HPO annotations can be well captured. Since the HPO annotation matrix is binary, we choose to use Non-negative Matrix Factorization (NMF). NMF has proved to be effective for sparse problems in the field of bioinformatics [13–16]. Based on our above observations, we propose an NMF-based framework called HPOAnnotator by which to predict missing protein-HPO annotations. In essence, the key idea of our model is to factorize the HPO annotation matrix into two non-negative low-rank latent matrices, which correspond to the respective latent feature spaces of proteins and HPO terms. In addition, the graph Laplacian on PPI networks is performed to exploit their intrinsic geometric structure. Co-annotations and the hierarchical structure of HPO are also incorporated to measure HPO semantic relationships.

We have experimentally validated the performance of HPOAnnotator by comparing it with the three network-based approaches, which will be reviewed in the related work. The proposed model was tested on the latest large-scale HPO data with around 300000 annotations. Experimental results clearly demonstrated that HPOAnnotator outperformed the competing methods under two scenarios: cross-validation and independent test. It indicates that a low-rank approximation and network information are effective for pair prediction. Furthermore, our case studies further provide evidence for the practical use of HPOAnnotator. Note that, the work presented in this paper is the extension of our previous work AiProAnnotator [17] (AiPA for short). The main difference between the two methods is that HPOAnnotator can seamlessly combine multiple rather than single PPI networks and then benefit from them.

## Related work

As mentioned before, we can group the existing approaches to HPO annotations into two categories: feature-based and network-based ones.

Two well-known methods of feature-based approaches are PHENOstruct [9] and Clus-HMC-Ens [18]. Clus-HMC-Ens applies the decision tree ensembles, while PHENOstruct (the extension of GOstruct which was designed to predict GO annotations) relies on the Structural Support Vector Machine (SSVM). Together with HPO annotations (i.e., labels) of each protein, a feature-based method normally accepts feature vectors as the input of a classifier. The trained classifier is then used to make a prediction. The above procedure is the same for both two categories of approaches. Additionally, it is worth noting that PHENOstruct and Clus-HMC-Ens were originally developed for GO but then applied to HPO annotation prediction. In this sense, the difference between HPO annotations and GO annotations has not been fully taken into account by researchers.

Relying on two networks of protein-HPO annotations and the hierarchy of HPO (or Network of HPO, called NHPO) with an optional PPI Network (hereafter PPN), the network-based approaches make predictions. The assumption behind them is that two nodes in a network should share some similarities, particular for those well-connected nodes who have more similarities. In the following, we review the three methods as representatives of network-based approaches, all of which are compared against our proposed approach in the experiments.

### Bi-random walk

Bi-Random Walk (BiRW) [19, 20] has been demonstrated as a useful method for the bi-network prediction problem. BiRW performs random walks on the Kronecker product graph between PPN and NHPO in a way that they can be combined effectively for the protein-phenotype association prediction. The random walks iteratively performed by BiRW follow the equation:

1 $$  \mathbf{Y}_{t} = \alpha \mathbf{P} \mathbf{Y}_{t-1} \mathbf{G} + (1 - \alpha) \mathbf{ \widetilde{Y} }  $$

where *α*>0 is a decay factor, **P** and **G** are the normalized PPN and NHPO matrix, respectively. **Y**_*t*_ is the estimation of associations at iteration *t*, and $\mathbf { \widetilde {Y} }$ denotes the initial annotations in the training data. By introducing BiRW to capture the circular bigraphs patterns in the networks, the model can unveil phenome-genome associations over time.

### Dual label propagation model

The label propagation-based algorithm has been successfully applied to predict phenotype-gene associations in various forms [21, 22]. With the following objective function, label propagation assumes that proteins should be assigned to the same label, if they are connected in a PPN:

2 $$ \begin{aligned}  \Psi(\mathbf{y}) &= \theta \sum_{i,j=1}^{n_{p}} \bar{\mathbf{S}}^{p} (y_{i} - y_{j})^{2} + \sum_{i} (y_{i} - \widetilde{y}_{i})^{2} \\ &= \theta \mathbf{y}^{T} \mathbf{L}_{S} \mathbf{y} + (1- \theta) \lVert \mathbf{y} - \widetilde{\mathbf{y}} \rVert^{2} \end{aligned}  $$

where $\bar {\mathbf {S}}^{p}$ is a normalized PPN defined as $\bar {\mathbf {S}}^{p} = \mathbf {D}^{-\frac {1}{2}}\mathbf {S}^{p}\mathbf {D}^{-\frac {1}{2}}$, and **D** is a diagonal matrix with the row-sum of **S**^*p*^ on the diagonal entries. Equation 2 can be rewritten as follows:

3 $$  \Psi(\mathbf{Y}) = \theta \text{tr} (\mathbf{Y}^{T} \mathbf{L}_{S} \mathbf{Y}) + (1 - \theta) \lVert \mathbf{Y} - \mathbf{\widetilde{Y}} \rVert_{F}^{2}  $$

where tr(·) denotes the trace of matrix, ∥·∥_*F*_ denotes the Frobenius norm, and **L**_*S*_ is the normalized graph Laplacian matrix of $ \bar {\mathbf {S}}^{p}$ defined as $\mathbf {L}_{S} = \mathbf {I} - \bar {\mathbf {S}}^{p}$.

The Dual Label Propagation model (DLP) [23] extends the label propagation model by adding two smoothness terms. The first term imposes the smoothness in a PPN such that interacting proteins tend to be associated with the same HPO term. The second term imposes the smoothness in NHPO in a way that the connected phenotypes (parent-child pair) are encouraged to be associated with the same protein. The objective function of DLP is given as:

4 $$  \Psi(\mathbf{Y}) = \lVert \boldsymbol{\Omega} \odot (\mathbf{Y} - \mathbf{\widetilde{Y}}) \rVert_{F}^{2} + \beta \text{tr}(\mathbf{Y}^{T} \mathbf{L}_{S} \mathbf{Y}) + \gamma \text{tr} (\mathbf{Y} \mathbf{L}_{G_{Y}} \mathbf{Y}^{T})  $$

where *β*,*γ*≥0 are tuning parameters, **L**_*S*_ and $\mathbf {L}_{G_{Y}}$ encode the PPN and NHPO information, respectively. ***Ω*** is the binary indicator matrix that selects only the known associations to be penalized, and ⊙ denotes Hadamard product (a.k.a entrywise product).

### Ontology-guided group lasso

The last method to be reviewed is Ontology-guided Group Lasso (OGL) [24]. It uses an ontology-guided group norm for HPO, rather than the graph regularizer in DLP. By combining label propagation and an ontology-guided group lasso norm derived from the hierarchical structure of HPO, OGL updates estimation, according to the following objective function:

5 $$ {\begin{aligned} \Psi (\mathbf{Y}) = \lVert \boldsymbol{\Omega} \odot (\mathbf{Y} - \mathbf{\widetilde{Y}}) \rVert_{F}^{2} + \beta \text{tr}(\mathbf{Y}^{T} \mathbf{L}_{S} \mathbf{Y}) + \gamma \sum_{i=1}^{n_{p}} \sum_{g \in \mathcal{G}_{Y}} r_{g}^{Y} \lVert \mathbf{Y}_{(g)i} \rVert_{2} \end{aligned}}  $$

where *β*,*γ*≥0 are balancing factors. $r_{g}^{Y}$ is the group weight for group *g*. **Y**_(*g*)*i*_ selects the group members of group *g* from the *i*-th column of **Y**, and the smoothness is imposed through the *ℓ*_2_-norm group lasso (∥·∥_2_) among the members for the consistent prediction within the group. A notable difference between OGL and our model is that the estimated matrix is not factorized into low-rank matrices.

One of the biggest drawbacks of network-based methods is that data sparseness has a significant impact on the performance. As mentioned before, the current HPO annotations are quite sparse. In addition, all of the network based-methods suffer the heavy computational burden, as they accept a large-scale protein-HPO annotation matrix as an input directly.

## Methods

### Notation

Let $\phantom {\dot {i}\!}\mathbf {Y} \in \{ 0, 1 \}^{N_{p} \times N_{h}}$ be a protein-HPO annotation matrix, where *N*_*p*_ and *N*_*h*_ are the number of proteins and HPO terms, respectively. If protein *i* is annotated by an HPO term *j*, then **Y**_*ij*_=1, and 0 otherwise. We define $\phantom {\dot {i}\!}\mathbf {S}^{p_{k}}$ (*k*=1,2,⋯,*t*) be the networks for proteins, namely PPNs, where *t* is the total number of networks. $\mathbf {S}^{p_{k}}_{i,j}$ represents the strength of the relationship between protein *i* and protein *j* in the *k*-th PPN. Similarly, let **S**^*h*^ be the network of HPO terms which is generated from an ontology structure and co-annotations, and $\mathbf {S}^{h}_{i,j}$ is the similarity value between term *i* and term *j*. Our goal is to estimate $\hat {\mathbf {Y}}$ given **Y**, $\phantom {\dot {i}\!}\mathbf {S}^{p_{k}}$ and **S**^*h*^.

### Our proposed method

#### Preprocessing: generating a network from HPO

The network of HPO terms, or NHPO, is derived by measuring the similarity between two HPO terms in a hierarchy. We adopt the measure proposed in [25]. Having been extensively used in natural language processing, this metric defines the semantic similarity between two labeled nodes by counting the co-occurrence frequency in a corpus.

Specifically for HPO, the semantic similarity between two terms *s* and *t* is defined as:

6 $$ \mathbf{S}^{h}_{s,t} = \frac{2 \cdot I(\text{mca}(s,t))}{I(s) + I(t)}   $$

where *I*(*s*)= log(*p*(*s*)) and $p(s) = \frac {\text {count}(s)}{N_{p}}$. Here, count(*s*) denotes the number of proteins annotated by term *s* and mca(*s,t*) is given as follows:

$$ \text{mca}(s,t) = \arg \min_{k \in \mathrm{A}(s,t)} p(k) $$

where A(*s,t*) represents the set of all common ancestors of *s* and *t*.

The weight of the edge between nodes *s* and *t* in NHPO is exactly the similarity score. The larger the number of annotated proteins shared by *s* and *t*, the higher their similarity score is. It is more likely to happen when the common ancestor of *s* and *t* is located closely. This means that **S**^*h*^ considers both the co-annotations of two HPO terms and their distance in a hierarchical structure.

#### Non-negative matrix factorization

The aim of Non-negative Matrix Factorization (NMF) is to find two low-rank matrices with all non-negative elements by approximating the original input matrix. In fact, the latent factors that underlie the interactions are captured. Mathematically, the input matrix ${\mathbf {Y} \in \mathbb {R}^{N_{p} \times N_{h}}_{+}}$ is decomposed into two rank-*K* matrices, $\mathbf {U} \in \mathbb {R}^{N_{p} \times K }_{+}$ and $\mathbf {V} \in \mathbb {R}^{N_{h} \times K}_{+}$. Then, finding **U** and **V** can be done by minimizing the reconstruction error which is defined as:

7 $$ J = \lVert \mathbf{Y} - \mathbf{U} \mathbf{V}^{T} \rVert_{F}^{2}, \text{ s.t.} \mathbf{U} \geq 0, \mathbf{V} \geq 0   $$

Generally, the *ℓ*2 (Tikhonov) regularization is imposed to Eq. (7) so as to alleviate overfitting of **U** and **V**.

Since there are unknown (missing) entries in **Y**, we encode the missingness with a masking matrix $\phantom {\dot {i}\!}\mathbf {W} \in \{0, 1\}^{N_{p} \times N_{h}}$. If the annotation between protein *i* and HPO term *j* is missing, we set **W**_*ij*_=0. Otherwise, we set **W**_*ij*_=1, meaning that the element **Y**_*ij*_ is observed. Accordingly, **W** is also plugged as an extra input into our model. Together with the *ℓ*2-norm regularization terms, the objective function is refined as follows:

8 $$ \begin{aligned} J_{\text{NMF}} =& \lVert \mathbf{W} \odot (\mathbf{Y} - \mathbf{U} \mathbf{V}^{T}) \rVert_{F}^{2} \\&+ \lambda (\lVert \mathbf{U} \rVert_{F}^{2} + \lVert \mathbf{V} \rVert_{F}^{2}), \text{ s.t.} \mathbf{U} \geq 0, \mathbf{V} \geq 0  \end{aligned}  $$

where *λ* is a regularization coefficient.

The unobserved protein-HPO associations are completed by multiplying two factor matrices, or concretely, $\hat {\mathbf {Y}} = \mathbf {U} \mathbf {V}^{T}$.

#### Network regularization

Once we obtain the similarity matrix of HPO, **S**^*h*^, we can regularize **V** with the help of it. The basic idea is to impose smoothness constraints on the phenotype-side factors; that is

9 $$  \begin{aligned} & \frac{1}{2} \sum_{i,j} \mathbf{S}^{h}_{i,j} \lVert \mathbf{V}_{i} - \mathbf{V}_{j} \rVert^{2} \\ =~&\text{tr} (\mathbf{V}^{T} (\mathbf{D}^{h} - \mathbf{S}^{h}) \mathbf{V}) \\ =~&\text{tr} (\mathbf{V}^{T} \mathbf{L}^{h} \mathbf{V}) \end{aligned}  $$

where **V**_*i*_ is the *i*-th row vector of **V**, **D**^*h*^ is a diagonal matrix whose diagonals are the node degrees, and **L**^*h*^=**D**^*h*^−**S**^*h*^ is the graph Laplacian of **S**^*h*^. Actually, the term is exactly the vanilla graph regularizer.

For proteins, multiple PPNs are derived from diverse data sources with heterogeneous properties. In this way, for a collective of PPNs $\phantom {\dot {i}\!}\mathbf {S}^{p_{k}} (k = 1, \cdots, t)$, their regularizer is imposed as

10 $$ \sum_{k=1}^{t} \text{tr}(\mathbf{U}^{T} \mathbf{L}^{p_{k}} \mathbf{U}),   $$

where $\mathbf {L}^{p_{k}} = \mathbf {D}^{p_{k}} - \mathbf {S}^{p_{k}} $ is the graph Laplacian of $\phantom {\dot {i}\!}S^{p_{k}}$, and $\phantom {\dot {i}\!}\mathbf {D}^{p_{k}}$ is the degree matrix.

Minimization of graph-based regularization terms will lead to the learned data representations (**U** and **V**) that respect the intrinsic geometrical structure of original data spaces ($\phantom {\dot {i}\!}\mathbf {S}^{p_{k}}$ and **S**^*h*^). Note that such standard graph regularization has already been used in a variety of applications [26].

#### Model formulation

By combining (8), (9) and (10), our model is formulated as follows:

11 $$ {\begin{aligned} & \min_{\mathbf{U} \geq 0, \mathbf{V} \geq 0} \lVert \mathbf{W} \odot (\mathbf{Y} - \mathbf{U} \mathbf{V}^{T}) \rVert_{F}^{2} + \lambda (\lVert \mathbf{U} \rVert_{F}^{2} + \lVert \mathbf{V} \rVert_{F}^{2}) \\&\quad+ \alpha \sum_{k=1}^{t} \text{tr} (\mathbf{U}^{T} \mathbf{L}^{p_{k}} \mathbf{U}) + \beta \text{tr} (\mathbf{V}^{T} \mathbf{L}^{h} \mathbf{V}) \end{aligned}}  $$

where *α* and *β* are regularization coefficients to strike a balance between the reconstruction error and graph smoothness.

#### Model optimization

Notice that the objective function defined in Eq. (11) is biconvex with respect to **U** and **V**. A very regular but effective procedure for fitting is Alternating Least Square (ALS), which alternately optimizes one of the variables by fixing the others as constants until convergence.

We first hold **U** fixed and derive the updating rule of **V**. The objective function of **V** can be written as:

12 $$  J(\mathbf{V}) = \lVert \mathbf{W} \odot (\mathbf{Y} - \mathbf{U} \mathbf{V}^{T}) \rVert_{F}^{2} + \lambda \lVert \mathbf{V} \rVert_{F}^{2} + \beta \text{tr} (\mathbf{V}^{T}\mathbf{L}^{h}\mathbf{V})  $$

Accordingly, the derivative of *J*(**V**) with respect to **V** is

13 $$  \frac{\partial J(\mathbf{V})}{\partial \mathbf{V}} = -2 (\mathbf{W} \odot \mathbf{Y})^{T}\mathbf{U} + 2(\mathbf{W} \odot \mathbf{U}\mathbf{V}^{T})^{T}\mathbf{U} + 2\lambda \mathbf{V} + 2\beta \mathbf{L}^{h}\mathbf{V}  $$

Taking the Karush-Kuhn-Tucker (KKT) complementary condition, we obtain

14 $$ [(\mathbf{W} \odot \mathbf{U}\mathbf{V}^{T})^{T}\mathbf{U} - (\mathbf{W} \odot \mathbf{Y})^{T}\mathbf{U} + \lambda \mathbf{V} + \beta \mathbf{L}^{h} \mathbf{V}]_{ij} \mathbf{V}_{ij} = 0  $$

Now let us rewrite **L**^*h*^=**L**^*h*+^−**L**^*h*−^, where we have **L**^*h*+^=(|**L**^*h*^|+**L**^*h*^)/2 and **L**^*h*−^=(|**L**^*h*^|−**L**^*h*^)/2. The multiplicative update rule of **V** is then:

15 $$  \mathbf{V}_{ij} \longleftarrow \mathbf{V}_{ij} \sqrt{ \frac{ (\mathbf{W} \odot \mathbf{Y})^{T}\mathbf{U} + \beta \mathbf{L}^{h-}\mathbf{V} } {(\mathbf{W} \odot \mathbf{U}\mathbf{V}^{T})^{T}\mathbf{U} + \lambda \mathbf{V} + \beta \mathbf{L}^{h+}\mathbf{V}} }  $$

Note that the problem given by (11) is symmetric in terms of **U** and **V**. Therefore, the derivation of the updating rule of **U** is simply the reverse of the above case. Precisely, we have

16 $$  \mathbf{U}_{ij} \longleftarrow \mathbf{U}_{ij} \sqrt{ \frac{ (\mathbf{W} \odot \mathbf{Y}) \mathbf{V} + \alpha \sum_{k=1}^{t}(\mathbf{L}^{p_{k}-} \mathbf{U}) }{(\mathbf{W} \odot \mathbf{U}\mathbf{V}^{T}) \mathbf{V} + \lambda \mathbf{U} + \alpha \sum_{k=1}^{t} (\mathbf{L}^{p_{k}+} \mathbf{U})} }  $$

#### Training algorithm

**Fig. 2:**
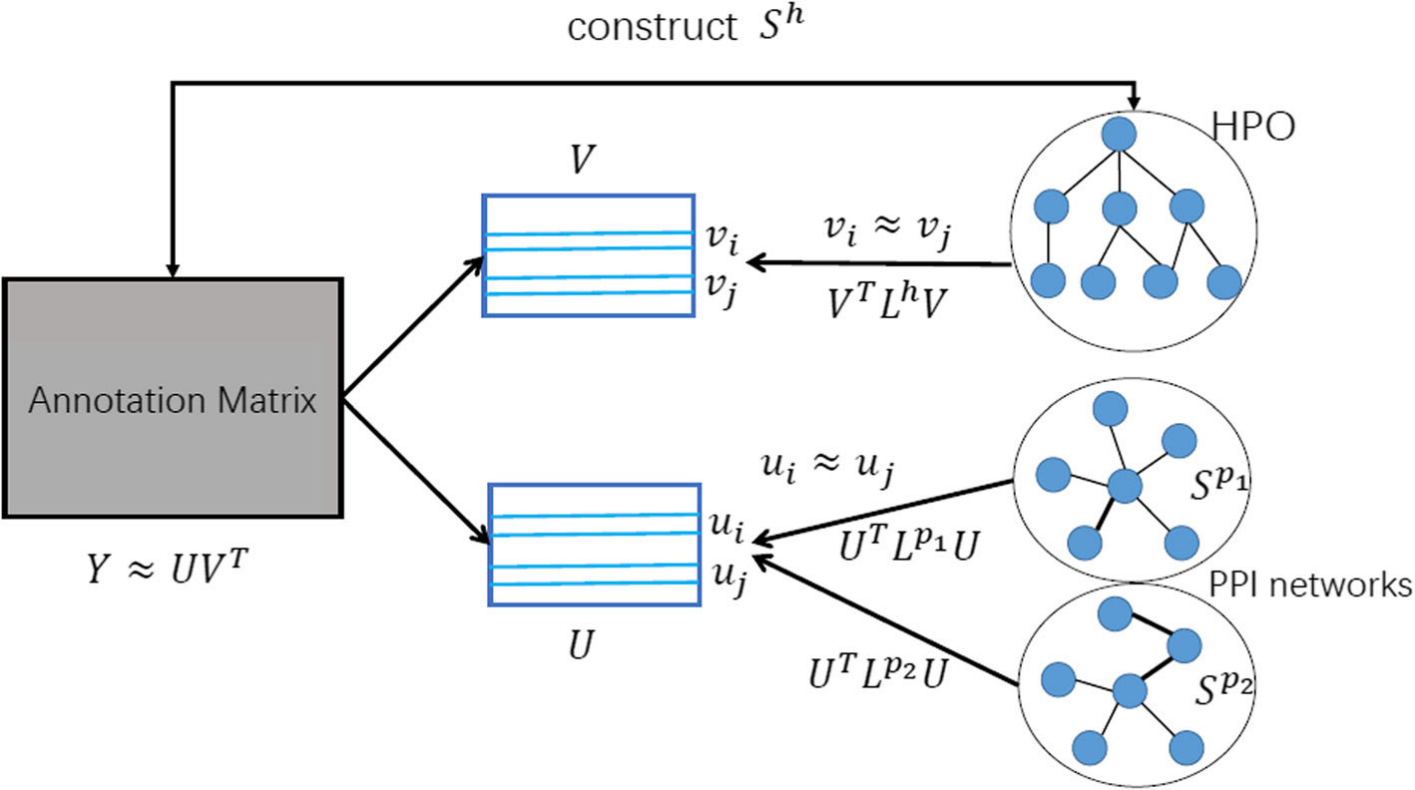
The framework of HPOAnnotator


We describe the overall framework of HPOAnnotator in Fig. 2. The procedure of our optimization process is presented in Algorithm 1. The optimization was implemented based on the MATLAB code provided by [26].

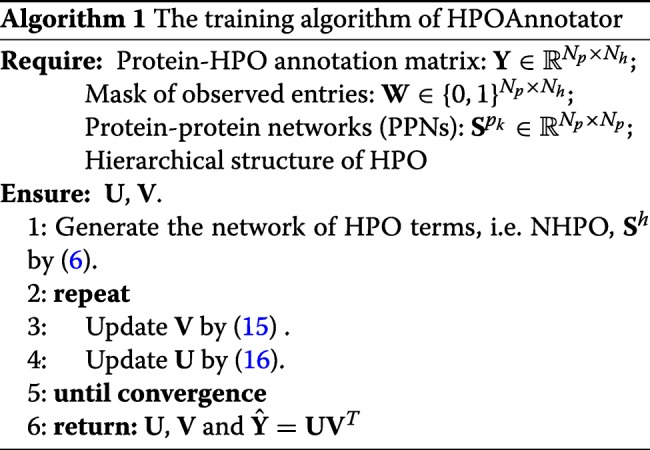


## Results

### Data

#### HPO annotations

Two HPO annotation datasets released by June 2017 and December 2017 were downloaded from the official HPO website (https://hpo.jax.org/). For the sake of brevity, we call them Data-201706 and Data-201712 in the following, respectively. The true-path-rule is applied here to propagate annotations, and only HPO terms with at least one related protein remains. Table 1 lists the statistics of the two datasets.

**Table 1:** Statistics of two datasets: Data-201706 and Data-201712

Dataset	Data-201706	Data-201712
#Proteins	3,459	3,644
#HPO terms	6,407	6,642
#Leaves of HPO	4,092	4,274
#Annotations	284,621	317,443
Ave. #annotations per protein	82.28	87.11
Ave. #annotations per HPO term	44.42	47.79

**Fig. 3:**
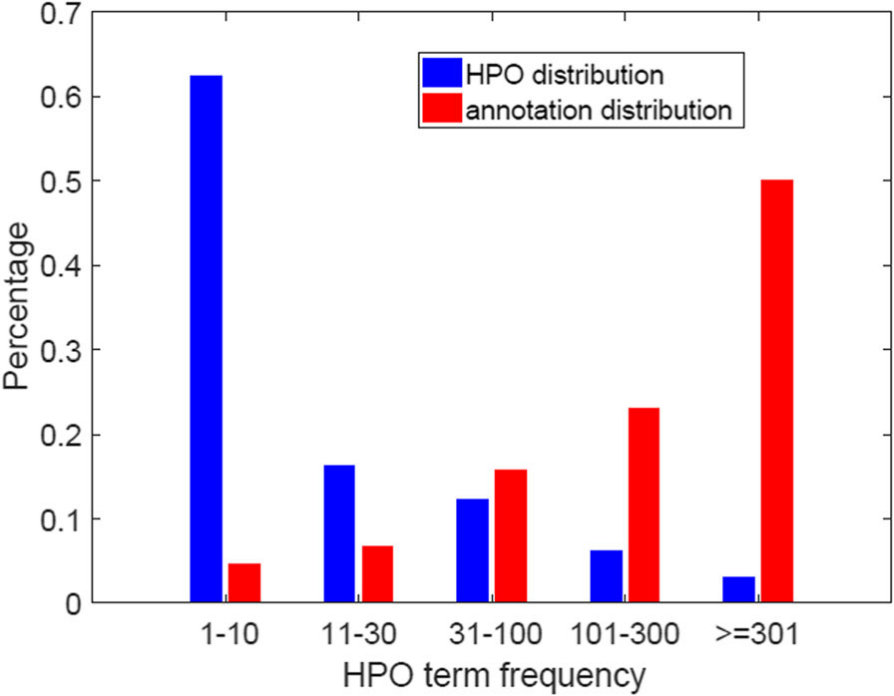
HPO terms are divided into five groups according to the number of proteins they annotate. The number of HPO terms per group (the left-hand side of each group) and the total number of annotations per group (the right-hand side of each group) are shown for Data-201706

According to the number of proteins annotated, we separated the HPO terms into five groups: 1 to 10, 11 to 30, 31 to 100, 101 to 300, and more than 300. Figure 3 shows the percentage of HPO terms and corresponding annotations over five groups in Data-201706.

#### NHPO (Network of HPO)

We downloaded the hierarchical structure of HPO from their official website.

#### PPN (Protein-Protein Network)

Four types of PPNs were used in our experiments; that is, STRING [27] (https://string-db.org/), GeneMANIA [28] (http://genemania.org/data/), BioGRID [29] (https://downloads.thebiogrid.org/BioGRID), and Reactome [30] (https://reactome.org/download-data). Table 2 reports the statistics of these four networks. Note that STRING is the most famous PPI network, which was found very useful for predicting HPO annotations in [9]. It combines diverse data sources, including co-expression, co-occurrence, fusion, neighborhood, genetic interactions, and physical interactions, by assigning a confidence score to a certain pair of proteins for indicating its reliability.

**Table 2:** Statistics of PPNs of Data-201706

Dataset	#Annotations	#Connect-proteins
STRING	214,410	3,342
GeneMANIA	206,900	3,385
BioGRID	10,752	2,725
Reactome	970	1,051

#### A preliminary test on pairs of two HPO terms in NHPO: the correlation between the number of shared proteins and the average similarity

**Fig. 4:**
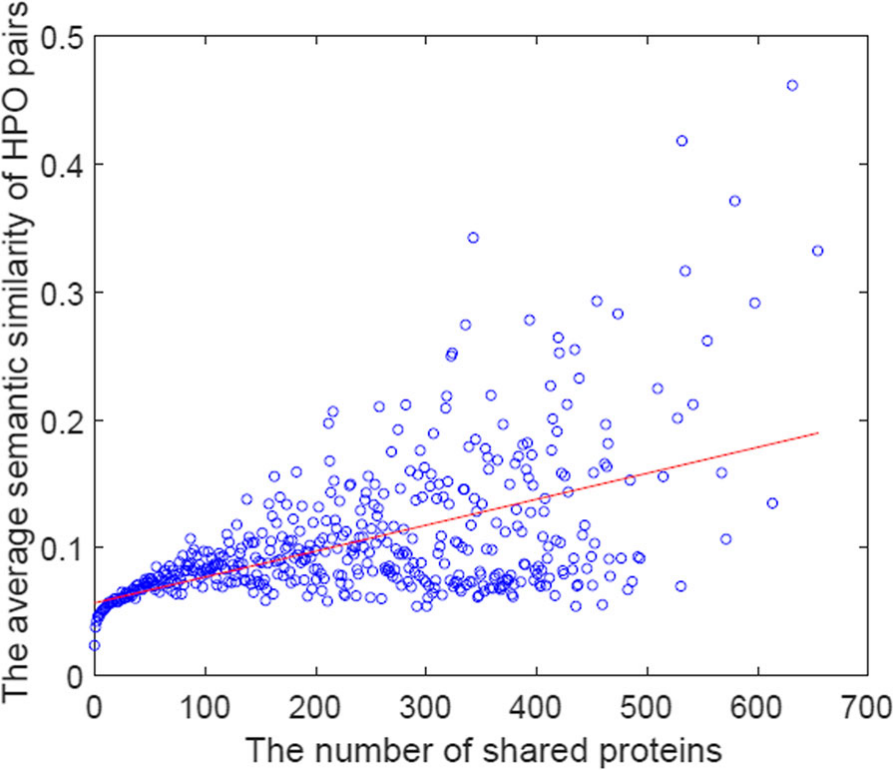
Each circle is a pair of two HPO terms in NHPO, with sharing the same numbers of proteins, say *M*. The *y*-axis is the average similarity score between two HPO terms over those proteins sharing the same *M*, and the *x*-axis is *M*, i.e., the number of shared proteins. The red line is fitted by a linear function

First, we grouped all pairs of two HPO terms (from NHPO), according to the number of proteins, say *M*, shared by the two HPO terms. For each group, we then computed the average similarity score (**S**^*h*^) by NHPO over those sharing *M* proteins. Finally, we plotted each group over the two-dimensional space of *M*× the average similarity score. Figure 4 shows the result. The similarity score is equal to the edge weight of NHPO. This means that this test would be evaluated on the consistency of the similarity with the number of shared proteins from each HPO term pair. There found some correlations between these two, which would be a positive support for using NHPO for HPO annotations.

#### A preliminary test on pairs of protein-protein edges in a PPN: correlations between the average similarity by a PPN and #shared HPO

**Fig. 5:**
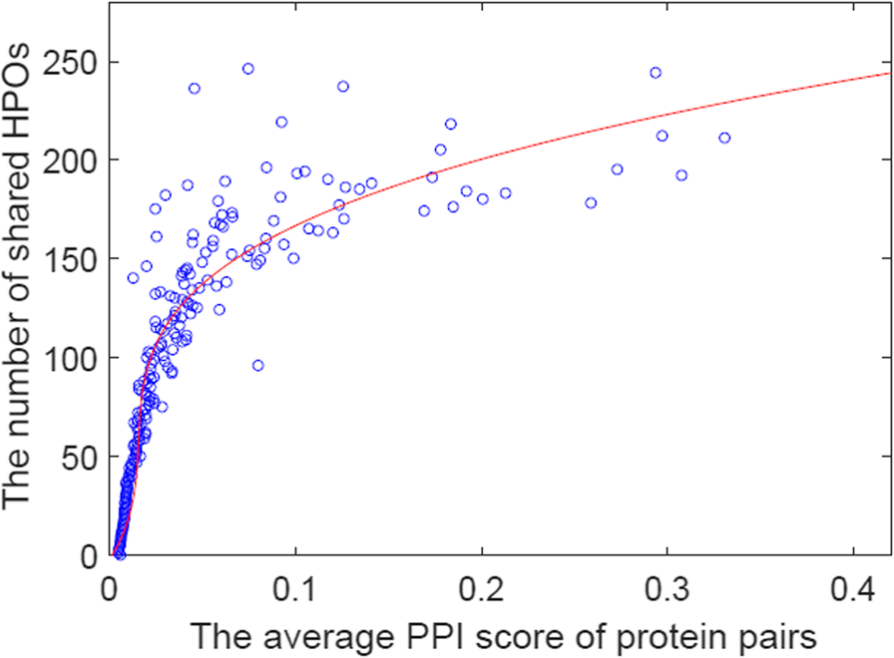
Each circle is a pair of two proteins in STRING PPN, with sharing the same numbers of HPO terms, say *K*. The *x*-axis is the average similarity score between two proteins over those HPO terms sharing the same *K*, and the *y*-axis is *K*, i.e., the number of shared HPO terms. The red line shows the trend, which is fitted by a polynomial function with the maximum degree of three

Considering the extensiveness, we chosen STRING as the research object. At first step, we grouped all pairs of two proteins, according to the number of their shared HPO terms, denoted as *K*. For each group, we then computed the average of similarity score (**S**^*p*^) of STRING PPN over those sharing the same number of HPO terms. Finally, we plotted each group over the two-dimensional space of the average score (similarity) ×*K*. Figure 5 shows the plotted results. The line in this figure shows that the polynomial trend line is fitted to the distributed points of the two-dimensional space. It shows a slightly positive correlation between the number of shared HPO terms and the average similarity score by a PPN. This observation validates the idea that the edges in a PPN may imply that proteins connected by the edges share the same HPO.

### Evaluation criteria

The performance is evaluated from three aspects.

**Annotation-centric measure** Each annotation (or a protein-HPO term pair) is viewed as one instance. The models are evaluated using Area Under the receiver operator characteristics Curve (AUC) [31]. Considering the sparseness of protein-HPO association matrix, we measure the Area Under the Precision-Recall curve (AUPR) as well.

**Protein-centric measure** AUCs (AUPRs) are calculated for each protein based on the corresponding predictive scores by all available HPO terms. Then the computed AUCs (AUPRs) are averaged over all proteins, resulting in micro-AUC (micro-AUPR).

**HPO term-centric measure** We think that the term-centric measure is important. Typical scientists or biologists focus first on a certain HPO term and are interested in obtaining genes/proteins, which can be annotated by the focused HPO term. The HPO term-centric measure can be computed in a total reverse manner of the protein-centric measure, with the following two steps: 1) AUCs (AUPRs) are first computed for each HPO term; and 2) The computed AUCs (AUPRs) are averaged over all HPO terms, which result in macro-AUC (macro-AUPR). In addition, we average the computed AUCs (AUPRs) over HPO terms at only leaves of the HPO hierarchical structure. We call the obtained AUC (AUPR) leaf-AUC (leaf-AUPR).

We further calculate the macro-AUCs (macro-AUPRs) for each of the five groups, which are generated by focusing on the number of annotations per HPO term (see Fig. 3). In total, (from annotation-, protein-, and HPO term-centric measures) we have the eight criteria to validate the performance.

### Experimental procedures

#### Parameter settings

Our approach is compared with three network-based methods: BiRW [20], DLP [23] and OGL [24] as described in related work. Besides, we take Logistic Regression (LR) as a feature-based baseline. Note that LR classifiers are trained on each single HPO term independently, and the features are built by concatenating association scores in PPNs together.

The parameter of BiRW is selected from {0.1,0.2,⋯,0.9}. Regularization coefficients (i.e., hyper-parameters) of DLP and OGL, *β* and *γ* are selected from {10^−6^,10^−5^,⋯,10^6^}. Note that the ranges of these parameters are specified by following [23]. Our model has four parameters: *K*, *α*, *β* and *λ*, which are determined by internal five-fold cross-validation, where the training data is further randomly divided into five folds (one for validation and the rest for training). The search ranges are as follows: {100,200} for *K*, {2^−3^,2^−2^,⋯,2^2^,2^3^} for *λ*, {2^−7^,2^−6^,⋯,2^6^,2^7^} for *α* and *β*.

There are several variants of our algorithm by changing the settings of hyper-parameters *α* and *β*. We also evaluate each of them as comparison methods. The details are as follows.

1. **NMF:** *α*=0 and *β*=0
  Now the model is reduced to standard NMF, and the objective function is exactly the same as Eq. (8).
2. **NMF-PPN:** *α*≠0 and *β*=0
  Under this setting, there is no regularization term of NHPO, but PPN has. Thus, we term this model as NMF-PPN.
3. **NMF-NHPO:** *α*=0 and *β*≠0
  This setting is in contrast to NMF-PPN. That is, the regularization term of NHPO is kept, while that of PPN is not.

For the case of *α*≠0 and *β*≠0, there are two another variants depending on whether or not multiple PPNs are utilized.

1. **AiPA:** only one PPN is utilized
  It is proposed in our previous study [17], which can be regarded as a special case of HPOAnnotator because only single PPN of STRING is exploited.
2. **HPOAnnotator:** multiple PPNs are utilized
  It is our final model presented in this paper. All four PPNs are used, including STRING, GeneMANIA, BioGRID, and Reactome as described before.

#### Two evaluation settings

Under two different settings, we validate the performance of the compared methods from two viewpoints:

1. Cross-validation over Data-201706
  We conduct 5 ×5-fold cross-validation over all annotations on Data-201706. That is, we repeat the following procedure five times: all known annotations are divided randomly into five equal folds. The four folds are for training, while the remaining one is for test. After selecting the test annotation between protein *p* and HPO term *h*, all annotations between *p* and the descendants of term *h* in the hierarchical structure of HPO are removed from the training data, in order to avoid any overlaps between training data and test data. It means that we predict the annotation of protein *p* out of all unknown HPO terms, which is a fair and strict evaluation.
2. Independent test by using Data-201712
  HPO annotations are incomplete, due to various reasons, such as slow curation. The way of annotations might be changed over time. So we conduct additional several experiments other than regular cross-validation by using data obtained in different time periods. That is, the training data is obtained before June 2017. All annotations in Data-201706 are used for training, where an internal five-fold cross-validation is done for setting up parameter values. After training, annotations obtained from June to December 2017 are then used for testing.

### Experimental results

#### Predictive performance in cross-validation on Data-201706

Table 3 reports the scores of the eight criteria obtained by averaging over 5 ×5 cross-validation (25 runs in total) on Data-201706. In this experiment, we compare the nine methods in total. In particular, the four are existing methods (LR, BiRW, OGL and DLP), and another five are variants of our model (NMF, NMF-PPN, NMF-NHPO, AiPA and HPOAnnotator). Note that STRING is the only PPN utilized in NMF-PPN. From the table, it clearly shows that our five methods perform better than the four existing methods. For example, our four methods achieve around 0.5 to 0.56 in AUPR, while all the scores by the existing methods are less than 0.1. In fact, our five methods perform better than the existing methods with respect to all of the eight metrics. Thus, their performance differences are very clear. We can conclude that a low-rank approximation is useful for the HPO annotation problem. Furthermore, HPOAnnotator always outperforms other variants in eight conditions among our five methods. This indicates that network information is well incorporated into our formulation.

**Table 3:** The results of the eight criteria obtained by 5 ×5-fold cross-validation over Data-201706 for the nine competing methods in total

Method	AUC	AUPR	micro-AUC	micro-AUPR	macro-AUC	macro-AUPR	leaf-AUC	leaf-AUPR
LR	0.775	0.028	0.760	0.072	0.579	0.052	0.532	0.020
BiRW	0.875	0.066	0.826	0.096	0.732	0.056	0.597	0.031
OGL	0.785	0.051	0.776	0.078	0.603	0.034	0.536	0.014
DLP	0.902	0.073	0.875	0.100	0.736	0.094	0.659	0.055
NMF	0.961	0.496	0.900	0.273	0.753	0.139	0.701	0.089
NMF-PPN	0.963	0.525	0.902	0.281	0.756	0.142	0.703	0.089
NMF-NHPO	0.965	0.541	0.903	0.290	0.756	0.144	0.702	0.094
AiPA	0.970	0.559	0.905	0.295	0.760	0.146	0.705	0.096
HPOAnnotator	**0.971**	**0.562**	**0.907**	**0.296**	0.760	**0.152**	**0.706**	**0.097**

Method performs best in terms of this evaluation metric are in boldface

Table 4 lists the AUC scores obtained for five groups divided by the number of annotations. Again, the results reported in these tables demonstrate the same conclusion as that in Table 3. That is, HPOAnnotator outperforms all other methods in all of the cases. A similar trend is also shown in Table 5. In summary, our approach is capable of achieving the best performance for HPO annotations in terms of cross-validation.

**Table 4:** Macro-AUC obtained by 5 ×5-fold cross-validation over Data-201706 for the nine competing methods

Method	[1-10]	[11-30]	[31-100]	[101-300]	[ ≥301]
LR	0.526	0.553	0.633	0.735	0.755
BiRW	0.608	0.854	0.875	0.835	0.815
OGL	0.586	0.670	0.788	0.812	0.806
DLP	0.622	0.880	0.914	0.863	0.834
NMF	0.649	0.908	0.942	0.948	0.911
NMF-PPN	0.651	0.911	0.943	0.951	0.916
NMF-NHPO	0.653	0.919	0.946	0.947	0.919
AiPA	0.654	0.922	0.943	0.957	0.931
HPOAnnotator	**0.655**	**0.925**	**0.947**	**0.958**	**0.931**

Method performs best in terms of this evaluation metric are in boldface

**Table 5:** Macro-AUPR obtained by 5 ×5-fold cross-validation over Data-201706 for the nine competing methods

Method	[1-10]	[11-30]	[31-100]	[101-300]	[ ≥301]
LR	0.003	0.022	0.047	0.064	0.077
BiRW	0.023	0.119	0.164	0.175	0.155
OGL	0.005	0.024	0.056	0.087	0.132
DLP	0.028	0.135	0.182	0.223	0.182
NMF	0.032	0.204	0.362	0.470	0.428
NMF-PPN	0.032	0.206	0.365	0.479	0.440
NMF-NHPO	0.032	0.209	0.373	0.488	0.472
AiPA	0.033	0.216	0.369	0.500	0.482
HPOAnnotator	**0.034**	**0.219**	**0.375**	**0.510**	**0.487**

Method performs best in terms of this evaluation metric are in boldface

A noteworthy point is that our method works well for the HPO terms with a very small number of annotations, i.e., only one to ten annotations per HPO term. In fact, this situation is usually hard for a low-rank approximation. As HPOAnnotator has achieved the best performance, this implies that a low-rank approximation is useful for all types of groups including HPO terms with a very small number of annotations for HPO annotations.

#### The effectiveness of individual PPNs in cross-validation on Data-201706

By using NMF-PPN, we perform a set of experiments in order to identify the most effective PPN in terms of HPO predictions. To this end, we perform a series of experiments on NMF-PPN by using a single PPN as its input at a time. NMF-PPN with the four PPNs performs best as reported in Table 6. As shown in Table 6, we can conclude that STRING is the most useful PPN for predicting HPO annotations. By the way, Our model can take advantage of different PPNs to achieve the best performance.

**Table 6:** Performance of NMF-PPN with individual PPNs

Data scource	AUPR	micro-AUPR	macro-AUPR
STRING	0.525	0.281	0.142
GeneMANIA	0.523	0.280	0.143
BioGRID	0.517	0.280	0.140
Reactome	0.505	0.278	0.139
All	**0.545**	**0.283**	**0.145**

Results are for each PPN on the Data-201706. “All” means all four PPNs are used.

Method performs best in terms of this evaluation metric are in boldface

#### Computation times in cross-validation on Data-201706

The computation (training) times of the eight methods compared in the cross-validation are recorded, where the times are averaged over the total 25 runs (5 ×5 folds). The computation times on the same machine with the same settings are reported in Table 7. From the table, our four models run faster than the compared ones. In fact, they are more than eight times faster than OGL and DLP. The training data is updated periodically, thus the model must be trained by the updated data often. As such, this advantage of our models would make a difference. In addition, OGL and DLP need much more memory spaces than the compared methods.

**Table 7:** Training times of a single run in 5 ×5-fold cross-validation (average over 25 runs)

Method	Computation time
LR	∼3.5 hours
BiRW	∼1.5 hours
OGL, DLP	≥4 hours
NMF, NMF-PPN, NMF-NHPO, HPOAnnotator	∼30 minutes

#### Predictive performance in the independent test on Data-201712

Table 8 reports AUC obtained by the experiments conducted on independent data for the eight competing methods. Among the three existing methods, DLP achieves the best performance, with AUC of 0.8298. NMF outperforms DLP with AUC of 0.8527, while two variants of NMF with one network regularizer further achieves better performance with AUC of around 0.89. AiPA achieves 0.9187 of AUC with STRING PPN and NHPO. Most importantly, HPOAnnotator archives the best performance, with the AUC of more than 0.92.

**Table 8:** AUC obtained by independent test using Data-201712

Method	AUC
BiRW	0.7971
DLP	0.8298
OGL	0.7322
NMF	0.8527
NMF-PPN	0.8923
NMF-NHPO	0.8959
AiPA	0.9187
HPOAnnotator	**0.9231**

Method performs best in terms of this evaluation metric are in boldface

As Table 9 reports, seven out of the 30 highest ranked predicted annotations are validated to be true according to Data-201712 which is released later. For example, protein Q02388, encoded by gene COL7A1, is actually annotated by HPO term HP:0001072 (*Thickened skin*). But we fail to find it in the data released by December 2017. Another example is protein Q9UBX5. According to Data-201706, it has no relationship with HPO term HP:0012638 (*Abnormality of nervous system physiology*). But this record occurs in the later release of the data.

**Table 9:** Seven true predictions out of the top 30 results (by HPOAnnotator) among all newly added annotations

Rank	Protein ID	Protein name	Gene name	HPO ID	HPO name
2	Q02388	Collagen alpha-1(VII) chain (Long-chain collagen) (LC collagen)	COL7A1	HP:0001072	Thickened skin
7	Q9UBX5	Fibulin-5	FBLN5 DANCE, UNQ184/PRO210	HP:0012638	Abnormality of nervous system physiology
17	Q9H5I5	Piezo-type mechanosensitive ion channel component 2 (Protein FAM38B)	PIEZO2	HP:0000422	Abnormality of the nasal bridge
19	O43175	D-3-phosphoglycerate dehydrogenase (3-PGDH) (EC 1.1.1.95) (2-oxoglutarate reductase) (EC 1.1.1.399) (Malate dehydrogenase) (EC 1.1.1.37)	PHGDH	HP:0000366	Abnormality of the nose
24	Q02388	Collagen alpha-1(VII) chain (Long-chain collagen) (LC collagen)	COL7A1	HP:0000962	Hyperkeratosis
26	Q04656	Copper-transporting ATPase 1 (EC 3.6.3.54) (Copper pump 1) (Menkes disease-associated protein)	ATP7A	HP:0002650	Scoliosis
27	P43026	Growth/differentiation factor 5	GDF5 BMP14, CDMP1	HP:0005622	Broad long bones

These seven annotations were not in the training data (Data-201706), but found in the latest release (Data-201712)

As the highest-ranked new annotation found by our model, HP:0001072 is known to also annotate another ten proteins, O43897, P07585, P08123, P08253, P12111, P20849, P20908, P25067, P53420, and Q13751, based on Data-201706. We find that their similarity scores with Q02388 in STRING are more than 0.9. It indicates that their interactions between Q02388 and those ten proteins in PPNs imply a high possibility of annotating Q02388 by HP:0001072. In summary, the number of these examples have demonstrated both the effectiveness and necessity of introducing PPI networks for unknown HPO annotations prediction.

#### Validating false positives

As mentioned before, seven of the top 30 correct predictions from our model have already been found in the December 2017 release version of HPO annotations. Due to the fact that a curation process on HPO annotations is normally slow, we believe that there may be more false positives among our top ranked predictions. In order to validate our assumption, we first select the rest of the top 10 predictions that have not been found in the December 2017 HPO data. Using a protein name (or its coding gene name) and an HPO term name as a query for online search engines, we then check the relevant literature and diseases for each false prediction. Finally, we manually extract the information from the retrieved papers containing supporting evidence that suggest a particular false positive to be correct in fact. Using this manual process, we find evidence for another two predictions. Table 10 lists the PubMed IDs of the relevant literature, the relevant diseases names, and the detailed evidence for each pair of the found gene/protein-HPO term. The results strongly indicate that the performance of HPOAnnotator is under-estimated, which is caused by the incompleteness of the current gold standard.

**Table 10:** Validation of false positives in the top 10 ranked predictions

Gene name	Protein	HPO ID	HPO name	PubMed ID	Disease	Evidence
SH3TC2	Q8TF17	HP:0001315	Reduced tendon reflexes	PMID: 14574644	Charcot-Marie-Tooth disease 4C (CMT4C)	"Demyelinating neuropathies are characterized by severely reduced nerve conduction velocities (less than 38 m/sec), segmental demyelination and remyelination with onion bulb formations on nerve biopsy, slowly progressive distal muscle atrophy and weakness, *absent deep tendon reflexes*, and hollow feet. By convention autosomal recessive forms of demyelinating *Charcot-Marie-Tooth disease* are designated CMT4."
FOXG1	P55316	HP:0001263	Global developmental delay	PMID: 19578037	Rett syndrome congenital variant (RTTCV)	"*Rett syndrome*is a severe neurodevelopmental disorder representing one of the most common genetic causes of *mental retardation* in girls. The classic form is caused by MECP2 mutations. In two patients affected by the congenital variant of Rett we have recently identified mutations in the *FOXG1 gene* encoding a brain specific transcriptional repressor, essential for early development of the telencephalon."

#### A typical example of demonstrating the performance of HPOAnnotator

To further demonstrate the performance of our proposed method for predicting HPO annotations, we here present the different predictions made by the four methods for a typical example, protein P23434. As listed in the last row of Table 11, this protein has 10 annotations. It is interesting to note that the number of correctly predicted HPO terms gradually increases from the first row to the fourth row. Again, this indicates that network information is effective for improving the performance of predicting HPO annotations.

**Table 11:** Predicted HPO terms of P23434 (gene name: GCSH) by our four methods based on NMF

Method	Predicted HPO terms	Correct
NMF	HP:0002079, HP:0001276, **HP:0000007**, HP:0007256, HP:0003287, **HP:0000718**, HP:0000729, HP:0002167, HP:0001268, HP:0002360	2
NMF-NHPO	**HP:0000007**, HP:0002079, **HP:0001250**, HP:0001276, **HP:0000718**, HP:0000729, HP:0012444, HP:0007256, HP:0002360, HP:0000478	3
NMF-PPN	**HP:0000007**, HP:0001276, HP:0007256, HP:0000729, **HP:0000718**, HP:0000478, HP:0003287, HP:0001268, **HP:0001298**, **HP:0001250**	4
HPOAnnotator	**HP:0000007**, **HP:0001250**, **HP:0001298**, HP:0000005, HP:0000707, **HP:0000718**, HP:0002167, **HP:0000711**, HP:0000924, HP:0000234	5
True	HP:0000007, HP:0000711, HP:0000718, HP:0001250, HP:0001298, HP:0001522, HP:0002086, HP:0002795, HP:0100247, HP:0100710	

Correctly predicted HPO terms are in boldface

#### Performance comparisons focusing on Organ abnormality

Most of the existing models are evaluated on separate sub-ontologies. However, considering only part of the ontology may lose entire network information. Such information can connect proteins or HPO terms that are even beyond the boundaries of two or more subontologies in the network space. As such, we do not conduct the experiments on separate sub-ontologies. Instead, we focus on the major sub-ontology, Organ abnormality (the part under HP:0000118), with 6370 HPO terms, 3446 proteins and 269420 annotations in total according to Data-201706. A 5 ×5-fold cross-validation has been conducted by following the same splitting strategy as before. Table 12 reports the scores of the eight evaluation criteria obtained by all compared methods. The results clearly show that the performance differences among the seven cases are subtle. For example, HPOAnnotator achieves the best performance with respect to all evaluation measure except for leaf-AUC. Comparing NMF-Organ and NMF-PPN-Organ in terms of AUC, we can find that network information can help to improve the performance to a certain extent. Nonetheless, the use of both networks of PPN and NHPO might not be so effective in this scenario. Besides, it seems that the performance improvement is quite limited when we consider the whole ontology rather than individual sub-ontologies. Tables 13 and 14 list the evaluation scores of Macro-AUC and Macro-AUPR over the five HPO term groups, respectively. The trend is similar to that presented in Table 12. Again, the results show no notable difference among the compared methods.

**Table 12:** Performance results on Data-201706 focusing on the sub-ontology Organ abnormality

Method	AUC	AUPR	micro-AUC	micro-AUPR	macro-AUC	macro-AUPR	leaf-AUC	leaf-AUPR
NMF-Organ	0.955	0.507	0.883	0.250	0.745	0.127	0.682	0.077
NMF-PPN-Organ	0.962	0.555	0.889	0.276	0.755	0.144	0.701	0.091
NMF-NHPO-Organ	0.962	0.535	0.888	0.264	0.756	0.141	0.702	0.089
NMF-All	0.956	0.512	0.884	0.258	0.755	0.129	0.685	0.083
NMF-PPN-All	0.962	0.553	0.889	0.273	0.755	0.143	0.698	0.089
NMF-NHPO-All	0.962	0.556	0.889	0.274	0.755	0.144	0.699	0.090
HPOAnnotator-All	**0.963**	**0.559**	**0.891**	**0.278**	**0.759**	**0.146**	0.702	**0.094**

The first three rows of methods with “Organ” are trained by HPO terms on Organ abnormality, while the others with “All” are trained by considering all sub-ontologies.

Method performs best in terms of this evaluation metric are in boldface

**Table 13:** Macro-AUC obtained by focusing on Organ abnormality

Method	[1-10]	[11-30]	[31-100]	[101-300]	[ ≥301]
NMF-Organ	0.645	0.897	0.924	0.945	0.922
NMF-PPN-Organ	0.654	0.921	0.943	0.956	0.934
NMF-NHPO-Organ	0.652	0.926	0.942	**0.958**	**0.936**
NMF-All	0.645	0.906	0.939	0.941	0.912
NMF-PPN-All	0.651	0.924	0.941	0.954	0.919
NMF-NHPO-All	0.650	0.928	0.940	0.953	0.935
HPOAnnotator-All	**0.655**	**0.929**	**0.946**	0.955	**0.938**

The three rows with “Organ" use only organ abnormality for training, while the others with “All" take all sub-ontologies for training.

Method performs best in terms of this evaluation metric are in boldface

**Table 14:** Macro-AUPR obtained by focusing on Organ abnormality

Method	[1-10]	[11-30]	[31-100]	[101-300]	[ ≥301]
NMF-Organ	0.030	0.190	0.355	0.478	0.446
NMF-PPN-Organ	0.033	0.205	0.371	**0.495**	**0.486**
NMF-NHPO-Organ	0.033	0.207	0.369	0.490	0.485
NMF-All	0.031	0.193	0.363	0.477	0.449
NMF-PPN-All	0.032	0.204	0.370	0.486	0.460
NMF-NHPO-All	0.032	0.209	0.373	0.482	0.462
HPOAnnotator-All	**0.035**	**0.212**	**0.374**	0.493	0.485

The three rows with “Organ" use only organ abnormality for training, while the other four rows with “All" take all sub-ontologies for training.

Method performs best in terms of this evaluation metric are in boldface

## Discussion and Conclusion

In this paper, we have presented an approach that uses a low-rank approximation to solve the problem of the large-scale prediction of HPO annotations for human proteins. In particular, network information is used to regulate such an approximation. The network information can be derived from both sides of annotations, i.e., PPI networks, and a hierarchical structure of an ontology. In essence, we provided a low-rank approximation solution to the optimization problem of matrix factorization with a network-derived regularization. Extensive experiments on the current HPO database have been conducted to validate the effectiveness of our approach. Experimental results clearly demonstrated the good performance of the proposed method under various settings, including cross-validation, independent test, analysis on the major sub-ontology Organ abnormality, and detailed case studies. The results have validated the good effectiveness as a result of using network information and ontology hierarchical structure as regularization and a low-rank approximation for HPO predictions, even for predictions on HPO terms with a very small number of known annotations.

Overall, the four important findings can be concluded from the experimental results: 1) a low-rank approximation works quite well for a large-scale HPO annotations prediction; or more generally, for multi-label classification, even for predicting labels with an extremely small number of labeled instances; 2) a hierarchical ontology structure is very useful as side information for improving the performance of a low-rank approximation; 3) PPI networks from different sources play an important role in predictions; and 4) multiplicative parameter update of a low-rank approximation (matrix factorization) is time-efficient, with around eight times faster than network-based approaches that need the huge memory because of using the original annotation matrices directly.

## Data Availability

Not applicable.

## Abbreviations

AiPA: AiProAnnotator
ALS: Alternating least square
AUC: Area under the receiver operator characteristics curve
AUPR: Area under the precision-recall curve
BiRW: Bi-random walk
CAFA: Critical assessment of functional annotation
DAG: Directed acyclic graph
DLP: Dual label propagation
GO: Gene ontology
HPO: Human phenotype ontology
KKT: Karush-kuhn-tucker
LR: Logistic regression
NHPO: Network of HPO
NMF: Non-negative matrix factorization
OGL: Ontology-guided group lasso
PPI: Protein-protein interaction
PPN: Protein-protein network
SSVM: Structural support vector machine
